# Prophylaxis of Invasive Fungal Infection in Neonates: A Narrative Review for Practical Purposes

**DOI:** 10.3390/jof9020164

**Published:** 2023-01-26

**Authors:** Giulia Ferrando, Elio Castagnola

**Affiliations:** Pediatric Infectious Diseases Unit, IRCCS Istituto Giannina Gaslini, 16147 Genova, Italy

**Keywords:** IFD (invasive fungal disease), preterm neonates, low birth weight neonates, neonatal intensive care unit (NICU), prophylaxis

## Abstract

*Candida albicans* is the most frequent cause of invasive fungal disease in preterm and/or low birth weight neonates, followed by *Candida parapsilosis*, whilst infections from other species are rare. Considering the severity of the disease, associated with poor clinical signs and diagnostic difficulties, primary prophylaxis becomes relevant. This paper summarizes the pathogenesis and clinical presentation of invasive candidiasis in neonates, focusing on prophylaxis. For late onset invasive disease, e.g., those occurring after the 3rd (or 7th according to some definitions) day of life possible approaches are the use of fluconazole, recommended in case of weight <1000 g or <1500 g if the local incidence of invasive candidiasis is higher than 2%, or the use of nystatin (for patients < 1500 g). Micafungin must be used in case of colonization by *Candida auris*, or in centers with a high prevalence of this pathogen. Concurrently, correct management of the central venous catheter and isolation procedures, with special regard to patients colonized by resistant strains, are fundamental. Other approaches such as reduced use of H_2_ blockers and broad-spectrum antibiotics (e.g., 3rd generation cephalosporins or carbapenems) and promotion of breast feeding proved useful. Reduction of early-onset infections (those occurring in the first 3 days of life) can also be obtained by treating maternal vulvo–vaginal candidiasis, which can represent a fastidious problem during pregnancy. In this case, topic azoles (the only recommendable treatment) can represent a kind of “prophylaxis” of early neonatal candidiasis. However, it must always be remembered that prophylaxis reduces the risk of invasive candidiasis but can not completely eliminate its occurrence, with the parallel risk of selecting for antifungal-resistant strains. Clinicians must maintain a high level of suspicion to start an appropriate therapy and strict epidemiological surveillance to identify the occurrence of clusters and the appearance of strains resistant to prophylaxis.

## 1. Introduction

Preterm and/or low birth weight neonates (Table 1) [1,2] represent a population that can need intensive treatment with long periods of hospitalization in a neonatal intensive care unit (NICU), where they are subjected to central vascular catheterization, parenteral nutrition, broad-spectrum antibacterial agents (e.g., third-generation cephalosporins and carbapenems) and other therapies (e.g., anti-acids), and can also require mechanical ventilation and/or surgery. Moreover, preterm infants present an immature and impaired adaptative immune response characterized by immunotolerance [1]. Finally, some very rare congenital disease, e.g., severe combined immunodeficiency, can be associated with an increased risk of infections, especially invasive fungal diseases in newborns [3]. 

All these conditions can be present at the same time or in sequence and are associated with an increased risk of infectious complications that can be classified according to time in early-onset (within 3rd day of life), late-onset (4th–60th day of life) and very late (>60th day of life) [4]. 

Invasive candidiasis is one of the most feared infections in this patients’ population because of the risk of long-term sequelae or death [5]. Birth weight and gestational age can be used to stratify patients’ risk of invasive fungal disease (IFD) since its incidence is inversely proportional to gestational age and weight [6]. 

*Candida* is a large group of more than 200 fungal species [1] and is a part of the normal microbiota of skin, bowel, and vagina, therefore, infants born via vaginal delivery are more likely to be colonized with *Candida* at birth as compared with infants born via cesarean section. *Candida albicans* is common in the female genitourinary tract and therefore can cause early post-natal colonization (and rarely infection) of the newborn; on the other hand, non-*albicans Candida* can also be present in the hospital environment and furniture with risk of nosocomial transmission mainly by healthcare workers hands [7] with infections due to this species generally occurring later after birth [8]. 

Indeed, colonization is a necessary but not sufficient condition to develop invasive infection. After colonization, favored by the previously listed conditions, *Candida* can penetrate into the bloodstream, with possible deep organ localizations [8,9] through several ways: vascular access [8,9] following hub or skin colonization [8,9] or injection of contaminated fluids; gastrointestinal mucosa, damaged by mucositis, abdominal surgery in the presence of necrotizing enterocolitis (NEC) or intestinal hypoperfusion associated with extracorporeal circulation for cardiac surgery, ischemic or toxic events [6,10,11]; respiratory tract, since the colonization of oropharynx and airways may cause candidemia in ventilated patient [12,13]; urinary tract, especially in presence of severe malformations [1].

Anyway, *Candida albicans* is the most frequent cause of IFD in neonates (66%), followed by *Candida parapsilosis* (31%) [3], while other species, such as *Candida tropicalis*, *Candida glabrata*, and *Candida krusei* are less frequent and together represent about 3% of IFD in neonates [14].

Clinical presentation is often not specific, with signs such as lethargy, apnea, or cardiorespiratory failure. Generally, there could be no correlation between the site of invasive mycosis and symptoms, even if a clinical picture of severe sepsis due to candidemia can be observed in 10% of cases, with 10.8% mortality in children under one year of age [15]. Isolated fungemia is the most frequent diagnosis of invasive candidiasis in neonates, but also central nervous system localization (meningoencephalitis) is quite frequent in these patients. Meningitis or encephalitis can often be neurologically asymptomatic, and sometimes, the cerebrospinal fluid examination could be normal. Other possible, less frequent sites of localizations are endocarditis, endophthalmitis, liver and spleen abscesses, osteoarthritis, intestine with perforation secondary to candidemia, kidneys usually associated with urinary obstruction from fungal bezoar, especially in patients affected by urinary tract malformation [1]. *Candida* pneumonia is generally hematogenous, and lung infection due to fungal aspiration is very rare and can be observed only in neonates with reflux and oro-pharyngeal secretions aspirations [16]. Finally, in addition to damages associated with deep organ localizations, candidemia has been associated with long-term sequelae such as preterm retinopathy or neurological impairment.

Diagnosis of invasive candidiasis is not easy, either directly by means of cultures or indirectly by means of biomarkers or polymerase chain reaction [17].

Therefore, if we consider the severity of the disease associated with poor clinical signs and the diagnostic difficulties, primary prophylaxis becomes a more than reasonable option.

## 2. Pharmacological Prophylaxis with Antifungal Drugs

Fluconazole, at the dosage of 3–6 mg/kg twice weekly (orally or intravenously) is considered the first choice for very low birth weight neonates [1,18,19]. Its administration should start from the second day of life and continue for a total of six weeks.. However, at least one clinical trial did not show any benefit on mortality in neonates < 750 g of birth weight [20], and no effect on the incidence of invasive mycoses in extremely low birth weight neonates was also reported in a pre–post cohort study performed in two tertiary care NICUs [21]. Fluconazole prophylaxis in neonates weighing 1000–1500 g can be adopted if the local incidence is higher than 2%

Administration of low-dose fluconazole for prophylaxis of invasive candidiasis could induce the selection of intrinsically resistant strains such as *C. krusei* or induce an increase of minimal inhibitory concentration and resistance in previously susceptible strains [22]. Only *ad hoc* epidemiological multicenter studies could give an answer to this question. At present while some centers have reported no effect on the incidence of intrinsically resistant strains [23,24] others have reported an increase in the proportion of fluconazole resistant *C. parapsilosis* [21,25,26]. Moreover, a further analysis of a randomized study on fluconazole prophylaxis showed an increase in minimal inhibitory concentrations (that anyway were still within the susceptibility range) of *Candida* colonizing patients during/after prophylaxis [27]. In this scenario, a preventive role of the policies of any single center regarding other prevention strategies can not be excluded. In neonates, fluconazole is generally well tolerated [28,29,30], but some concerns on the possible risk of neurodevelopmental impairment have been raised in the past [31,32]. Anyway, no negative effects in neonates under 750 g evaluated at 18 to 22 months of corrected age have been documented [20], and the evaluation at 8 to 10 years of life of neurodevelopmental status and quality of life of survivors from a randomized, placebo-controlled trial of fluconazole prophylaxis showed no differences in quality of life, behavior, communication, motor skills, and school performance [33]. 

A different consideration must be made for *Candida auris*. This is a yeast with high resistance to many antifungal drugs and its diffusion is increasing in many countries [34]. Because of its resistance profile, its presence is an exception to fluconazole prophylaxis: in colonized patients or in contexts with high rates of *C. auris* colonization, micafungin should be considered for antifungal prophylaxis in low-birth-weight and preterm neonates instead of fluconazole, at least until its presence is excluded [35,36]. At this point, a few words must be said about the risk of severe long-term hepatotoxicity induced by micafungin [37]. Pre-clinical studies in rats required by the Japanese Regulatory Agency, where the drug was initially developed, employed high doses (32 mg/kg) over 3–6 months, equivalent to an 8–17 years exposure in man; then following 3 months of exposure, multinucleated hepatocytes and altered hepatocellular foci were seen within 3 months and adenomas formed over a period of 21 months. Subsequently, when administered for a 6-month period, the time to the incidence of hepatic abnormalities increased and the formation of carcinomas was detected in female but not in male rats, suggesting a possible role of estrogens [38]. However, human administration does not achieve these cumulative doses and duration, and severe long-term hepatotoxicity has never been observed in humans [39,40]. 

In adults, it has been demonstrated that antifungal prophylaxis in cases of repeated intestinal surgery is associated with a reduction of invasive candidiasis in high-risk patients [41,42,43]. Fluconazole has been the most frequently administered drug, but also micafungin or caspofungin have been used [44,45]. Unfortunately, no similar study has been performed in pediatrics and even less in neonates, especially those undergoing intestinal surgery because of NEC. Considering the severity of invasive candidiasis in this context, this should be a field for future studies.

Nystatin 100.000 UI q8h orally has been indicated as possible antifungal prophylaxis; in alternative to fluconazole [1]. However, this drug has been associated with potential damage of intestinal epithelium and increased risk of NEC, and can not be tolerated in children with clinical instability, gastrointestinal diseases, or feed intolerance. 

## 3. Other Prophylaxis, including Non-Pharmacological Approaches

Bovine lactoferrin, alone or in combination with the probiotic *Lactobacillus rhamnosus* GG has been demonstrated to reduce the first episode of late-onset sepsis in very low birth weight neonates [46], including candidiasis, apparently in newborns not receiving fluconazole prophylaxis [47]. However, these results have not been confirmed in a recent large randomized clinical trial [48].

Granulocyte colony-stimulating factor (G-CSF) has been proposed in neutropenic (granulocyte absolute count < 1500/μL) neonates to reduce the risk of infectious complications, but its administration had no significant effect on infection-free and overall survival [49]. Similar results were observed in patients treated with granulocyte–monocyte (GM)-CSF, with the aggravating circumstance that an analysis of general health outcomes performed 2 years after the study showed that neonates treated with GM-CSF had a marginally increase in the incidence of cough and signs of chronic respiratory disease, even if not associated to bronchodilator use or need for hospitalization for this reason [49].

The administration of probiotics has also been proposed to reduce infectious complications in neonates, including candidiasis [46]. However, their role in the prevention of IFD in neonates is not clear since optimal strain, dosage, and duration of treatment are unknown, and there are concerns about the possibility of contamination [1]. Indeed, a recent systematic review [50] has shown the possibility, albeit rare, of invasive infections due to probiotics, especially in patients with comorbidities, and it must be stressed that probiotic yeasts, such as *Saccharomyces boulardii/cerevisiae*, can be very difficult to treat and are resistant to fluconazole [51].

Finally, the correct application and management of central venous catheter (bundle) and isolation procedures are pivotal to reduce the risk of severe infectious complications in NICU, including candidiasis [1,52], even if there is no need of a different infection control policy compared with that adopted in other intensive care units. Other approaches, such as reduced use of H2 blockers and broad spectrum antibiotics (especially 3rd generation cephalosporins and carbapenems), and promotion of breast feeding can be useful for the prevention of invasive infections, including candidiasis [1,52]. Daily chlorhexidine bath has been suggested to reduce invasive candidiasis [1,52], and other infections as those due to *Staphylococcus aureus*. However, there are concerns on the use of this compound in preterm neonates because of the risk of skin irritation and potential for systemic absorption in the first 2 months of life, with consequent restriction of its use at least within the first month, even if adverse events have been rarely documented [53].

Maternal vulvo–vaginal candidiasis is a frequent, fastidious condition during pregnancy that can be associated with vertical transmission of *Candida*. Treatment of this condition can therefore represent a kind of “prophylaxis” of the (rare) early neonatal candidiasis. Topical azoles represent the only recommended option for this purpose [54] since boric acid and fluconazole during pregnancy have been associated with potential teratogenic effects [55,56]. Table 2 summarizes all the options for the prevention of invasive candidiasis in the NICU.

In conclusion, antifungal prophylaxis reduces, but unfortunately does not completely eliminate, invasive fungal infections in NICU. Likewise, invasive fungal infections can occur in infants receiving antifungal prophylaxis. Therefore, clinicians must maintain a high level of suspicion for the possible occurrence of this complication for a prompt start of an appropriate therapy, that in the first days is necessarily empirical, and strict epidemiological surveillance in order to identify the possible occurrence of clusters and/or the appearance of strains resistant to antifungal prophylaxis. 

## Figures and Tables

**Table 1:** Definition of preterm and low birth weight neonates.

	Definitions	
**Gestational Age**	Extremely preterm	Born < 28th gestational week
	Very preterm	Born 28th– < 32nd gestational week
	Moderate or late preterm	32nd– < 37th gestational week
**Birth Weigh**	Extremely low birth weight (ELBW)	<1000 g
	Very low birth weight (VLBW)	<1500 g
	Low birthweight (LBW)	<2500 g

**Table 2:** Invasive candidiasis in preterm, low birth weight neonates: possible prophylactic approaches.

Procedure	Indication	Comment
Fluconazole, oral-intravenous	Birth weight < 1000 g	Recommended, no effect on mortality in birth weight < 750 g
	Birth weight < 1500 g	Recommended in NICU with incidence of invasive candidiasis > 2%
Nystatin, oral	Birth weight < 1500 g	Recommended, concerns with possible gut damage and risk of NEC; difficult administration in case of clinical instability, gastrointestinal diseases, feeding difficulties
Micafungin	Birth weight < 1000 g and colonization by *Candida auris*	Potential risk of hepatotoxicity, no demonstration of long-term hepatic disease
G-CSF, GM-CSF	Neutropenia: absolute granulocyte count < 1500/mL	No demonstration of effectiveness
Bovine lactoferrin	Birth weight < 1500 g	Effectiveness not confirmed in recent large randomized clinical trial
Probiotics	Birth weight < 1500 g	Suggested increased effectiveness in combination with bovine lactoferrin; risk of invasive disease by probiotics with potential difficulties in anti-infective therapy because of drug resistance
Infection control measures	Patients admitted to NICU	Recommended; effective also in contrasting diffusion of (resistant) pathogens
CVC-bundle	Patients admitted to NICU	Recommended; reduced risk of CVC-related infections
Reduced use of H_2_ blockers and broad-spectrum antibiotics (mainly 3rd generation cephalosporins and carbapenems)	Patients admitted to NICU	Recommended; reduced risk of invasive infections
Promotion of breast milk	Patients admitted to NICU	Recommended; Reduced risk of invasive infections
Chlorhexidine bath	Patients admitted to NICU	Concerns about skin irritation and systemic absorption, especially in the first month of life
Topical azoles	Maternal candidiasis during pregnancy	Fluconazole and boric acid potentially teratogenic

NICU = neonatal intensive care unit. NEC = necrotizing enterocolitis. CVC = central venous catheter.

## Data Availability

Not applicable.
